# Linking microbial communities to rheumatoid arthritis: focus on gut, oral microbiome and their extracellular vesicles

**DOI:** 10.3389/fimmu.2025.1503474

**Published:** 2025-04-16

**Authors:** Jian Lu, Yi Wang, Jing Wu, Yusi Duan, Haifang Zhang, Hong Du

**Affiliations:** ^1^ Department of Clinical Laboratory, The Second Affiliated Hospital of Soochow University, Suzhou, Jiangsu, China; ^2^ Department of Laboratory Medicine, The Affiliated Guangji Hospital of Soochow University, Suzhou Mental Health Center, Suzhou, Jiangsu, China

**Keywords:** gut microbiome, oral microbiome, bacterial extracellular vesicles, rheumatoid arthritis, pathogenesis

## Abstract

Rheumatoid arthritis (RA) is a severe, chronic autoimmune disease affecting approximately 1% of the global population. Research has demonstrated that microorganisms play a crucial role in the onset and progression of RA. This indicates that the disruption of immune homeostasis may originate from mucosal sites, such as the gut and oral cavity. In the intestines of patients in the preclinical stage of RA, an increased abundance of *Prevotella* species with a strong association to the disease was observed. In the oral cavity, infections by *Porphyromonas gingivalis* and *Aggregatibacter actinomycetemcomitans* can mediate the production of anti-citrullinated protein antibodies (ACPAs), potentially contributing to RA pathogenesis. Nevertheless, no single bacterial species has been consistently identified as the primary driver of RA. This review will discuss the connection between gut and oral bacteria in the development of arthritis. Additionally, it explores the role of bacterial extracellular vesicles (bEVs) in inducing inflammation and their potential pathogenic roles in RA.

## 1 Introduction

Rheumatoid Arthritis (RA) is a clinically significant, refractory autoimmune disease, characterized by uncontrolled inflammation and cartilage destruction in the affected joints, eventually leading to joint dysfunction and deformity (1). Current therapeutic regimens include glucocorticoids, non-steroidal anti-inflammatory drugs (NSAIDs), disease-modifying anti-rheumatic drugs (DMARDs), and small molecule inhibitors. Although these medications can significantly ameliorate patients’ clinical symptoms, they are not curative.

Currently, more than 60% of RA patients do not achieve true remission, placing a substantial burden on social health and the economy. The pathogenesis of RA is complex, and its exact etiology remains unclear, with factors such as smoking, hormones, gut microorganisms, and infections all associated with RA development. At its core, RA is driven by autoimmunity, where the immune system mistakenly attacks self-tissues, particularly synovial joints. This autoimmune response is closely linked to microbiome dysregulation, which is pivotal in shaping immune tolerance and systemic inflammation. The interplay between autoimmunity and microbiome dysregulation provides a critical framework for understanding RA pathogenesis (2). Notably, the dysregulation of the gut and oral microbiota has emerged as a key contributor to RA pathogenesis, potentially through molecular mimicry, epitope spreading, and the activation of autoreactive T and B cells. Recent studies have shown that IgA antibodies against cyclic citrullinated peptides can be detected in RA patients several years before the disease onset. IgA antibodies are primarily found in mucous membranes, indicating that RA could potentially originate in areas such as the intestinal tract, oral cavity, and lung mucosa. Individuals with an elevated risk of developing RA are known to exhibit signs of persistent systemic and mucosal inflammation. Current research links the progression of mucosal dysbiosis, inflammatory processes, and the production of autoantibodies with subsequent phases in the emergence of systemic autoimmunity (3–6).

Antimicrobial drug therapy, such as minocycline or salazosulfadiazine, has proven effective in some RA patients, indicating a close association between the intestinal and oral microbiota and the onset and progression of RA (7, 8). Furthermore, certain medications that modulate intestinal microbiota homeostasis, including biotics (prebiotics, probiotics, and postbiotics) and some traditional Chinese medicine, have demonstrated significant efficacy in RA, further supporting the notion that microbial factors may be important pathogenic elements and therapeutic targets in the development and progression of RA (9–12). Therefore, Understanding the regulatory mechanisms of pathogenic microorganisms in the pathogenesis of RA is crucial for developing new therapeutic strategies and preventive measures. Intervening with relevant pathogenic microorganisms can potentially regulate immune system imbalances, reduce systemic inflammation, and block the development of RA. This study aims to summarize the pathogenic roles and mechanisms of these microorganisms in RA. It will cover both conventional and novel mechanisms, particularly focusing on how bacterial extracellular vesicles (bEVs) modulate the inflammatory response and contribute to the progression of RA.

## 2 Bacterial factors in RA development

### 2.1 Intestinal flora imbalance

The imbalance in gut microbiota plays an important role in the pathogenesis of autoimmune diseases (13, 14). In RA, studies have demonstrated that the gut microbiome of patients exhibits an ecological imbalance, characterized by specific microbial features associated with increased intestinal permeability, inflammatory cell infiltration, and the production of anti-citrullinated protein antibodies (ACPAs). In recent years, researchers have employed 16S rRNA and Next Generation Sequencing (NGS) technologies to conduct comprehensive studies on the gut microbiota of RA patients and healthy individuals. The study results demonstrated that the microbial composition in the gut of pre-clinical RA patients changed significantly, coupled with a marked reduction in microbial diversity. Notably, pre-clinical RA patients exhibited a pronounced enrichment of *prevotellaceae* (15, 16). Similarly, an increased abundance of *Prevotella copri* and a decreased abundance of *Bacteroides* species have also been observed in American patients with new-onset RA (17). This pattern has also been noted in Japanese and European cohorts (18). Nii and Maeda et al. revealed that *Prevotella copri* can induce cytokines associated with Th17 cells, such as IL-6 and IL-23, promoting arthritis development in mice. Moreover, this increase in *Prevotella* correlates with Th17 cell-mediated mucosal inflammation, consistent with its capacity to drive Th17 immune responses *in vitro* (19). Notably, anti-rheumatic drug-mediated disease remission was observed to increase microbial richness and diversity (20, 21).

Apart from *Prevotella*, the abundance of other gut bacteria has also been confirmed to change significantly in patients with RA. Jeong et al. underscored significant differences in microbial distributions from phylum to genus levels between healthy individuals and early RA patients. The phylum *Bacteroidetes* was enriched in early RA patients, whereas *Actinobacteria*, such as genus *Collinsella*, were more prevalent among healthy subjects (22). Zhang et al. observed an elevated presence of *Lactobacillus salivarius* in the gut, teeth, and saliva of RA patients, alongside a decrease in *Haemophilus* species (21). *Fusobacterium nucleatum (F. nucleatum)* was also significantly enriched in RA patients and positively correlated with inflammatory cytokines and disease activity, suggesting its potential involvement in RA inflammation (23). Notably, the intestinal microbiota in RA patients exhibits dynamic changes across different stages of the disease. *Collinsella aerofaciens*, which has been associated with severe arthritis in experiments, was significantly elevated in the early RA stage, potentially compromising gut barrier integrity and triggering clinical arthritis. In the later stages, specific microbes such as *Veillonella parvula*, *Eggerthella lenta*, and *Bifidobacterium longum* were elevated and associated with increased gut permeability and inflammation (24). Gut bacteriome alterations in RA are summarized in **Table 1**.

**Table 1:** Alterations in the bacteriome in RA.

Study object	Technology	Increased Taxa	Decreased Taxa	Ref.
Gut bacteriome				
RA patients and healthy controls	16S rRNA	*Prevotella*	*Bacteroides*	(17)
RA patients and healthy controls	16S rRNA	*Prevotella*	*/*	(18)
RA patients and healthy controls	Metagenome	*Prevotella, Bacteroides sartorii, Gardnerella, and Porphyromonas somerae*	*/*	(25)
RA patients and healthy controls	Metagenome	*Bacteroides, Clostridium asparagiforme, and Lactobacillus*	*Klebsiella pneumonia, Haemophilus, and Veillonella*	(21)
RA patients and healthy controls	qPCR	*Bacteroides and Prevotella*	*Clostridium leptum*	(26)
RA patients and healthy controls	16S rRNA	*Ruminococcus, Fusobacterium, Erysipelatoclostridium, and Mitochondria*	*/*	(23)
RA patients and healthy controls	16S rRNA	*Firmicutes, Actinobacteria, and Bifidobacterium dentium (RA II, RA III, RA IV); Collinsella aerofaciens (RA I); Veillonella parvula (RA III); Eggerthella lenta and Bifidobacterium longum (RA IV)*	*Bacteroides uniformis (RA II); Bacteroides plebeius (RA III and RA IV)*	(24)
RA patients and healthy controls	16S rRNA	*Lactobacillus*	*Faecalibacterium*	(27)
RA patients and healthy controls.	16S rRNA	*Escherichia-Shigella and Bacteroides*	*Lactobacillus, Alloprevotella, and Enterobacter*	(28)
RA patients and healthy controls	16S rRNA	*Eubacterium_hallii, Escherichia-Shigella, and Streptococcus*	*/*	(29)
RA patients and healthy controls	16S rRNA	*Ruminococcus, Fusobacterium, Erysipelatoclostridium, and Mitochondria*	*Muribaculaceae, Agathobacter, and Alloprevotella*	(23)
Female RA patients and healthy controls	16S rRNA	*Bacteroidetes*	*Actinobacteria*	(22)
Female RA patients and healthy controls	16S rRNA	*Bacteroides, Megamonas, and Oscillospira*	*Prevotella, Gemmiger and Roseburia*	(30)
RA and OA patients	16S rRNA	*Prevotella copri*	*Bacteroides and Bifidobacterium*	(31)
Pre-clinical RA patients and healthy controls	16S rRNA	*Prevotella* spp	*/*	(15)
Pre-clinical RA patients and first-degree relatives	16S rRNA	*Prevotella, Lactobacillus, Butyrivibrio, Ruminococcaceae, and Enterococcus*	*/*	(16)
Pre-clinical RA patients and healthy controls	16S rRNA	*Helicobacteraceae*, *Erysipelotrichaceae, and Ruminococcaceae*	*Bacteroidaceae, Barnesiellaceae, and Methanobacteriaceae*	(32)
Pre-clinical RA patients and healthy controls	16S rRNA	*Klebsiella, Escherichia, Eisenbergiella, and Flavobacterium*	*Fusicatenibacter, Megamonas, and Enterococcus*	(33)
Pre-clinical RA patients and healthy controls	16S rRNA	*Lactobacillus, Streptococcus*, *and Akkermansia*	*Bacteroides and Faecalibacterium*	(34)
Pre-clinical RA patients and healthy controls	16S rRNA	*Lactobacillus, Raoultibacter*	*Ruminococcus, Pseudomonas, and Ruminiclostridium*	(35)
Oral bacteriome				
RA patients and healthy controls	16S rRNA	*Treponema, Porphyromonas, Prevotella, and Veilonella*	*Streptococcus, Gemella, and Planobacterium*	(36)
Early RA patients and healthy controls	16S rRNA	*Porphyromonas and Fusobacterium genera*	*/*	(37)
Pre-clinical RA patients and new-onset or chronic RA	16S rRNA	*Porphyromonadacae*	*/*	(38)
RA patients in different stages of disease activity	16S rRNA	*Treponema and Absconditabacteriales (remission);* *Porphyromonas (low disease activity);* *Staphylococcus (low disease activity)*	*/*	(39)
RA patients and control subjects with or without periodontitis	qPCR	*Aggregaticbacter actinomycetemcomitans*	*/*	(40)
RA patients and control subjects with periodontitis	16S rRNA	*Prevotella, Aggregaticbacter actinomycetemcomitans,and Parvimonas micra*	*/*	(41)
Pre-clinical RA patients and healthy controls	16S rRNA	*Prevotella*	*Defluviitaleaceae and Neisseria oralis*	(42)
RA patients and non-RA periodontitis group	16S rRNA	*Granulicatella, Veillonella, Megasphaera, and Fusobacterium nucleatum*	*Alloprevotella, Prevotella, Haemophilus, and Actinomyces*	(43)
RA patients and healthy controls	16S rRNA	*Cryptobacterium curtum, Atopobium* spp*, and Lactobacillus salivarius*	*Haemophilus, Aggregatibacter, and Cardiobacterium, Eikenella, and Kingella*	(21)
RA patients and healthy controls	16S rRNA	*Cryptobacterium curtum*	*Aggregatibacter, Gemella, Granulicatella, Haemophilus, Neisseria, and Streptococci*	(44)
Early RA patients and healthy controls	16S rRNA	*Prevotella, Veillonella*	*/*	(45)

### 2.2 Oral pathogenic bacterial infection

The initial inflammation in both RA and periodontitis is caused by the activation of intrinsic cells, monocyte macrophages, and dendritic cells. Both diseases exhibit enhanced bone resorption (46). The development of RA may accelerate periodontal tissue destruction, with the extent of periodontal damage being positively correlated with the severity of RA disease activity. Animal experiments have demonstrated that the development of periodontitis exacerbates the progression of RA (47). Notably, the elimination of periodontal lesions significantly attenuated RA disease progression, even in the absence of targeted treatment, suggesting that periodontal disease and RA may share similar pathogenic mechanisms (48, 49).

*Porphyromonas gingivalis (P. gingivalis)* is a pathogen that colonizes the oral cavity and is commonly associated with periodontal diseases. However, its pathogenic effects extend beyond the oral cavity. *P. gingivalis* can also contribute to the occurrence of various diseases such as Alzheimer’s disease, colon cancer, and diabetes via the digestive tract and bloodstream (50, 51). In RA, infection with *P. gingivalis* is closely associated with disease progression (36–38). Studies indicate that RA patients have a significantly higher likelihood of developing periodontal disease compared to non-RA patients, exhibiting more severe periodontal disease progression and a significant positive correlation with arthritis activity (52). Moreover, the majority of RA patients exhibit high levels of oral pathogenic bacterial DNA in their oral plaque and joint synovial fluid. *P. gingivalis* DNA is among the most easily detectable bacterial nucleic acids, showing a significantly increased detection rate compared to the control group (53, 54). Furthermore, it has been demonstrated that *P. gingivalis* can exacerbate T cell-driven arthritis through the induction of an antigen-specific Th17 response (55).

Citrullination, an essential post-translational protein modification, is primarily catalyzed by peptidylarginine deiminase (PAD) enzymes. PAD enzymes convert arginine residues on protein peptides to citrulline, which is targeted by RA-specific autoantibodies, thus stimulating self-reactive T cells and exacerbating inflammatory damage in the joints (56). *P. gingivalis*, a bacterium closely linked to the onset and progression of RA, possesses a unique enzyme known as *P. gingivalis* peptidylarginine deiminase (PPAD). This enzyme can induce citrullination in both self and host proteins. It is demonstrated that 33% of RA patients exhibit increased reactivity to anti-citrullinated PPAD (anti-cit-PPAD) antibodies prior to clinical disease onset. Furthermore, 77% of RA patients display anti-citrulline-specific immune responses to PPAD-derived peptides. Furthermore, studies have confirmed a correlation between the levels of anti-PPAD antibodies and ACPAs. These findings suggest that *P. gingivalis* may significantly contribute to RA pathogenesis and progression by promoting ACPAs production (57). In the collagen-induced arthritis (CIA) model, infection with *P. gingivalis* resulted in earlier onset, a more severe course, and enhanced disease progression. Moreover, compared to the wild-type strain, the ability of PPAD-deficient *P. gingivalis* to promote disease progression in RA model mice is significantly reduced (58). In addition to inducing protein citrullination, *P. gingivalis* may contribute to the progression of RA by affecting the intestinal immune system and gut microbiota composition. Fecal microbiota transplantation (FMT) from *P. gingivalis*-inoculated experimental arthritis mice resulted in more severe joint inflammation compared to FMT from control mice (59). *Aggregatibacter actinomycetemcomitans* (*A.a.*) is another pathogenic bacterium that plays a significant role in periodontal disease. This bacterium is notably enriched in the oral cavity of RA patients and has been associated with disease activity (40, 60). While *A.a.* does not have the ability to directly citrullinate proteins, it can induce high expression of PAD enzymes in neutrophils, thereby promoting self-antigen citrullination, triggering the formation of ACPAs, and exacerbating the autoimmune response in RA (61). Studies have demonstrated that patients infected with *A.a.* exhibit positive cyclic citrullinated peptide (CCP) antibodies and that *A.a.* infection is associated with the formation of these antibodies (60). However, the role and mechanisms of *A.a.* in the onset and development of RA still require further investigation. While early studies propose bidirectional interactions between periodontitis and RA, direct causal evidence linking *P. gingivalis* and *A.a.* induced periodontitis to RA progression in murine models is absent. Instead, findings predominantly support RA-driven exacerbation of periodontitis severity (62). This discrepancy may arise from experimental design limitations, such as inducing periodontitis after RA onset in animal studies, which could confound observational outcomes. Additionally, short experimental timelines and inherent differences between animal models and human disease complexity limit translatability. Thus, rigorous longitudinal studies are essential to clarify oral microbiota’s role in RA progression. Oral bacteriome alterations in RA are summarized in **Table 1**.

## 3 Pathogenic mechanisms

### 3.1 Metabolic dysregulation

Gut microbiota dysbiosis has been shown to alter the synthesis of microbial metabolites, leading to immune and metabolic imbalances. The gut microbiota produce Short-Chain Fatty Acids (SCFAs) such as acetate, propionate, and butyrate, and the modulation of these metabolites is closely linked to the onset of autoimmune diseases (63). In animal models of arthritis, SCFAs, particularly butyrate, have been shown to effectively inhibit osteoclast differentiation and prevent bone loss (64, 65). By influencing gene expression, butyrate can promote the differentiation of Treg cells, suppress Th17 cells, down-regulate pro-inflammatory cytokine production, and maintain immune homeostasis (66–68). In addition, butyrate also increases serotonin-derived 5-HIAA, directly activates regulatory B cells (Bregs), and inhibits germinal center B cell and plasmablast differentiation (65). Studies indicate that patients with RA exhibit a significant disruption in butyrate metabolism in the gut, leading to markedly decreased circulating butyrate levels (69). Notably, butyrate metabolism is associated with ACPAs production. Research suggests that the proportion of bacteria involved in butyrate metabolism negatively correlates with ACPAs titers and affected joint counts (66). Due to its ability to modulate immune responses via the Treg/IL-10/Th17 axis, butyrate administration mitigates joint inflammation and bone destruction in mice (70). Thus, gut microbiota-mediated butyrate metabolism might play a pivotal role in RA inflammation, suggesting that targeting butyrate metabolism could be a potential strategy for the clinical treatment of RA.

Additionally, abnormal tryptophan metabolism has been closely associated with the disruption of immune tolerance, potentially triggering the onset of autoimmune diseases such as RA (33, 71). Tryptophan, an essential amino acid, is primarily produced through the breakdown of food by *lactobacilli* and *bifidobacteria* and is further metabolized by intestinal bacteria into indole metabolites in the colon (72, 73). Research indicates that indole maintains epithelial cell structure and function, promotes goblet cell differentiation and mucin production, thereby enhancing intestinal barrier function and reducing inflammatory responses (74). Indole acetaldehyde and indole-3-acetic acid can induce CD4+ T cell differentiation into Treg cells through aryl hydrocarbon receptors. Additionally, indole-3-acetic acid can inhibit Th17 cell polarization (75, 76). Metabolomic analysis of fecal samples from patients with RA and healthy individuals revealed significantly decreased levels of downstream tryptophan metabolites in the feces of RA patients, including serotonin, xanthurenic acid, and 3-hydroxyanthranilic acid (3-HAA) (33). Xanthurenic acid exhibits immunosuppressive effects (77), while 3-HAA can inhibit macrophage inflammatory responses (78). Notably, tryptophan metabolite levels in the synovial fluid of RA patients are significantly lower than those in osteoarthritis patients (79), suggesting that tryptophan metabolism may play a crucial role in RA development, with intestinal dysbiosis potentially being a key factor in its onset and progression.

### 3.2 Molecular mimicry

Research indicates that some pathogenic microorganisms express homologous proteins similar to host proteins, leading to host immune imbalance under specific circumstances, which plays a critical role in the pathogenesis of autoimmune disorders such as RA. One aspect of molecular mimicry in RA involves the citrullination process, catalyzed by PPAD from *P. gingivalis*, a bacterium associated with periodontal disease and RA. PPAD catalyzes the citrullination of proteins such as vimentin, fibrinogen, and α-enolase, which are autoantigens in RA. This process results in the production of citrullinated proteins recognized by autoreactive T cells, leading to the generation of ACPAs, unique to RA (80). Additionally, microorganisms possess antigenic epitopes similar or identical to those of human cells. This similarity can trigger immune responses against these antigens upon infection. For instance, bacteria such as *Escherichia coli* and *Klebsiella pneumoniae* produce peptides like L-ASNase67-81, a segment of bacterial L-asparaginase, which can activate HLA-DRB10401-restricted T cells in early RA patients. This activation leads to the expression of CD154 and the production of cytokines such as IL-2, IL-17A/F, and IFN-γ, crucial in disease progression (81). Moreover, Peptides presented by HLA-DR from N-acetylglucosamine-6-sulfatase and filamin A show significant sequence similarity to epitopes from Prevotella sp. and Butyricimonas sp., which are targeted by T-cells and B-cells as auto-antigens in over half of RA patients. This similarity indicates a crucial role in RA development by triggering autoimmune responses to bacterial antigens (82). Similarly, shared sequences between Collinsella and DRB10401 suggest that Collinsella may also induce RA via molecular mimicry (20). These autoimmune epitopes are prominently found in bacterial species of the Firmicutes and Proteobacteria phyla, potentially having a greater impact on the disease in genetically susceptible individuals (83). Persistent colonization of bacteria with cross-reactive epitopes in hosts with high-risk Human Leukocyte Antigen (HLA) genes could lead to the sustained activation of auto-reactive T cells in the gut, contributing to RA development.

### 3.3 Altered intestinal permeability

Studies have indicated that disruption of the gut barrier, such as through apoptosis of intestinal epithelial cells caused by microbial infections, can lead to a pro-inflammatory environment and the differentiation of Th17 cells and other T helper cells. In a mouse model of RA, significant impairment of intestinal barrier function was observed before the onset of arthritis. Similarly, elevated serum markers associated with compromised intestinal barrier function in humans before RA onset are linked to an increased risk of developing RA (84). Additionally, there is a noted association between serological markers of intestinal permeability, disease activity, and response to biologic disease-modifying antirheumatic drugs (85). Mechanistically, dysbiosis in the gut microbiota can compromise intestinal mucosal integrity, activate the gut immune system, and trigger the migration of immune cells such as group 3 innate lymphoid cells (ILC3s), follicular helper T cells (Tfh cells), and mucosa-associated invariant T cells (MAITs) to the systemic circulation and joints, ultimately contributing to arthritis (86–90). These findings underscore a significant connection between compromised gut integrity and systemic inflammation in RA.

## 4 A novel pro-inflammatory factor: bEVs

Bacterial extracellular vesicles (bEVs) are spherical, double-layered vesicles secreted by bacteria, with diameters ranging from approximately 20 to 300 nm. bEVs can carry various effector molecules, including lipopolysaccharides, proteins, and nucleic acids, and participate in interactions between bacteria and host cells (91). Gram-negative and Gram-positive bacteria produce different types of bEVs because of differences in cell wall structure. bEVs secreted by Gram-negative bacteria are termed outer membrane vesicles (OMVs), while those released by Gram-positive bacteria are called cytoplasmic membrane vesicles (CMVs) (92). These bEVs can deliver their contents to target cells through receptor-ligand interactions, membrane fusion, or receptor-mediated endocytosis, thereby modulating the biological behavior of host cells. bEVs have been shown to induce a pro-inflammatory responses, thereby enhancing the host defense mechanism against infections. Additionally, bEVs can influence adaptive immune responses via antigen presentation and T-cell activation (**Table 2**). Studies indicate that bEVs are involved in the occurrence and progression of various autoimmune diseases (117).

**Table 2:** Pro-inflammatory effects of bEVs.

bEVs origin	Targeted cell/Tissue	Effect (↑ increase or enhancement)	Mechanism	Ref.
*Innate immune response*				
*Helicobacter pylori*	Gastric epithelial cells (AGS)	↑ proliferation ↑ IL-8 production	Not examined	(93)
*Staphylococcus aureus*	Lung epithelial cells (A549)	↑ IL‐8 and CCL2 production	NF-κB activation via TLRs and NOD2 signaling	(94)
*Clostridium botulinum*	Murine colonic epithelial cells (CMT-93) Human colorectal cancer cells (Caco-2)	↑ IL-6 and CCL2 production	MyD88/TRIF activation via TLR1/2 and TLR4	(95)
*Porphyromonas gingivalis*	Gingival keratinocytes and dendritic cells	↑ IL-6, TNF-α, and IL-1β production	PI3K-AKT activation via RgpA and EGFR interaction	(96)
	Human gingival epithelial cell (OBA-9)	↑ IL-6 and IL-8 production	MAPK and STING signaling	(97)
*Faecalibacterium prausnitzii*	Human colorectal cancer cells (Caco-2)	↑ IL-4, IL-8, and TNF-α production	Not examined	(98)
*Akkermansia muciniphila*	Murine macrophage (RAW 246.7)	↑ IL-6 production	Not examined	(99)
*Escherichia coli Nissle 1917*	Murine macrophage (RAW 246.7)	↑ proliferation, NO production, and acid phosphatase	Not examined	(100)
*Lactobacillus sakei*	Peyer’s Patch cells	↑ IgA production	TLR2 activation	(101)
*Bacteroides and Prevotella*	Murine lungs	↑ IL-17A and IL-17B, production ↑ Th17 cells	TLR-MyD88 adaptor signaling	(102)
*Legionella pneumophila*	Murine macrophage (THP-1)	↑IL-8, IL-6, IL-10, IL-1β, and TNF-α production	Via activation of TLR2, IRAK-1, and NF-κB	(103)
*Staphylococcus aureus*	Murine macrophages (NR-9456)	↑ IFN-β production	Via activation of endosomal TLRs (TLR3, TLR7, and TLR9)	(104)
*Escherichia coli*	Murine bone marrow-derived monocytes	↑ IL-1β production	Activation of the non-canonical inflammasome pathway	(105)
*Uropathogenic Escherichia coli*	Murine bone marrow-derived monocytes	↑ IL-1β production	NLRP3 inflammasome activation via mitochondrial apoptosis and potassium ion efflux	(106)
*Neisseria gonorrhoeae*				
*Pseudomonas aeruginosa*				
*Escherichia coli*	*In vivo*	↑ TNF-α and IL-6 production in serum and bronchoalveolar lavage fluid	Not examined	(107)
	Murine lung tissues	↑ neutrophils ↑ CXCL1expression	TLR4- and NF-κB-dependent manners	(108)
	Endothelial cells (HMEC-1)	↑ IL-8 production		
*Staphylococcus aureus*	Human keratinocytes	↑ IL-8 production	TLR2- and NF-κB-dependent manners	(109)
*Bacteroides thetaiotaomicron*	Murine bone marrow-derived monocytes	↑ TNF-α secretion	Not examined	(110)
*Adaptive immune responses*				
*Escherichia coli Nissle 1917 and EcoR12*	*In vivo*	↑ plasma IgG, IgA, and IgM ↑ Spleen Tc, NK, and NKT cells	Not examined	(111)
*Gut microbiota*	Gut epithelium (HT-29)	↑ T-cell-independent sIgA response	NF-κB activation-mediated APRIL, CCL28 and PIGR expression	(112)
*Akkermansia muciniphila*	Murine small intestine	↑ B cells and dendritic cells activation	Not examined	(113)
*Escherichia coli Nissle 1917*	Human Monocyte Derived-DCs	↑ Th1 responses	Not examined	(114)
*P. gingivalis and Treponema denticola*	Mouse bone marrow-derived dendritic cells	↑ Th17 responses	Not examined	(115)
*Escherichia coli*	*In vivo*	↑ CXCL10 and IFN-γ production	IFN-γ-dependent manner	(116)

### 4.1 The role of bEVs in promoting innate immune responses

Bacterial extracellular vesicles (bEVs) play a crucial role in host-microbe interactions by disrupting mucosal integrity and internalizing into various cell types, notably epithelial cells, which are primary contact points for bacteria. This interaction induces diverse immune responses, primarily through the activation of Toll-like receptors (TLRs) and other pathogen recognition receptors (PRRs) that recognize pathogen-associated molecular patterns (PAMPs), leading to immune activation. The inflammatory potential of bEVs was first observed when interleukin (IL)-8 was released from gastric epithelial cells stimulated by *Helicobacter pylori* bEVs (93). Additionally, *Staphylococcus aureus* can employ bEVs to transport lipoproteins, nucleic acids, and peptidoglycan, which are recognizable by host PRRs. This interaction has been demonstrated in human lung epithelial cells, where bEVs stimulation led to the secretion of cytokines IL-8 and CCL2, along with a strain-specific secretion of the pro-inflammatory cytokine IL-6 (94). Similarly, bEVs from various *Clostridium* sp*ecies* have been shown to enhance IL-6 and CCL2 expression in mouse colonic epithelial cells and human colorectal cancer cells (95). While TLRs signaling is a well-documented pathway, bEVs also promote inflammatory responses through the dissemination of toxins. For example, gingipains from *P. gingivalis* bEVs can induce the secretion of IL-6, IL-8, and TNF-α by interacting with the epidermal growth factor receptor within the lipid rafts of keratinocytes (96, 97).

As tissue-resident cells, macrophages are pivotal in intercepting bacterial extracellular vesicles (bEVs) that penetrate the mucosal barrier. Upon contact with the cellular surface or following phagocytosis, these bEVs activate various inflammatory pathways in macrophages, influenced by their specific cargos. Notably, macrophages employ Toll-like receptors (TLRs) to recognize bEVs, thereby initiating the release of inflammatory cytokines. Research by Yang et al. demonstrated that bEVs from *Bacteroides* and *Prevotella* species exacerbate bleomycin-induced lung fibrosis. This interaction involves bEVs binding to TLR4 and TLR2 on alveolar macrophages via their lipopolysaccharides (LPS) and lipoproteins, respectively, leading to the secretion of IL-17b, a key cytokine in this fibrosis model (102). Further studies with THP-1 macrophage-like cells have shown that bEVs from *L. pneumophila* activate TLR2 signaling, leading to the secretion of cytokines, including IL-8, IL-6, IL-10, IL-1β, and TNF-α (103). Beyond proteins, the nucleic acids present on or within bEVs can also engage TLRs. For instance, *Staphylococcus aureus* bEVs trigger TLR3, TLR7, and TLR9, inducing IFN-β expression in NR-9456 murine macrophages (104). Moreover, bEVs can initiate inflammasome activation, a fundamental inflammatory pathway, as seen with *Escherichia coli (E. coli)* strains expressing the virulence factor HlyF, which enhances IL-1β release and increases cell death through a non-canonical pathway (105, 118). Such inflammasome activation may also be stimulated by bEVs-induced mitochondrial dysfunction caused by pathogens such as uropathogenic *E. coli*, *P. aeruginosa*, and *Neisseria gonorrhoeae* (106). These findings underscore the diverse and intricate mechanisms by which bEVs, carrying varied cargos, mediate inflammation through both TLR signaling and inflammasome activation, highlighting the complex nature of bEV-mediated immune modulation.

The localized release of chemoattractant molecules is pivotal in recruiting neutrophils to control infections or participate in inflammation. However, the interaction between neutrophils and bacterial extracellular vesicles (bEVs) introduces further complexity to this immune response. Studies have demonstrated that bEVs from *E. coli*, when administered intraperitoneally, accumulate in various organs and lead to increased leukocyte infiltration, particularly neutrophils (107). Additional research revealed that these bEVs colocalize with endothelial cells in mice lungs, inducing the neutrophil chemoattractant CXCL1 expression. Complementary *in vitro* experiments with human endothelial cells demonstrated that the CXCL1 homolog, IL-8, is produced through TLR4 in an NF-κB-dependent manner (108). In skin, Staudenmaier et al. observed that bEVs from *Staphylococcus aureus* induce the release of IL-8 from human keratinocytes through NF-κB signaling, dependent on TLR2 (109). The intricate interaction highlights the multifaceted role of bEVs in modulating host immune responses and underscores the need for additional research to completely understand their mechanisms and implications in host-pathogen dynamics. The pro-inflammatory effects of bEVs on the innate immune response are summarized in **Table 2**.

**Figure 1:**
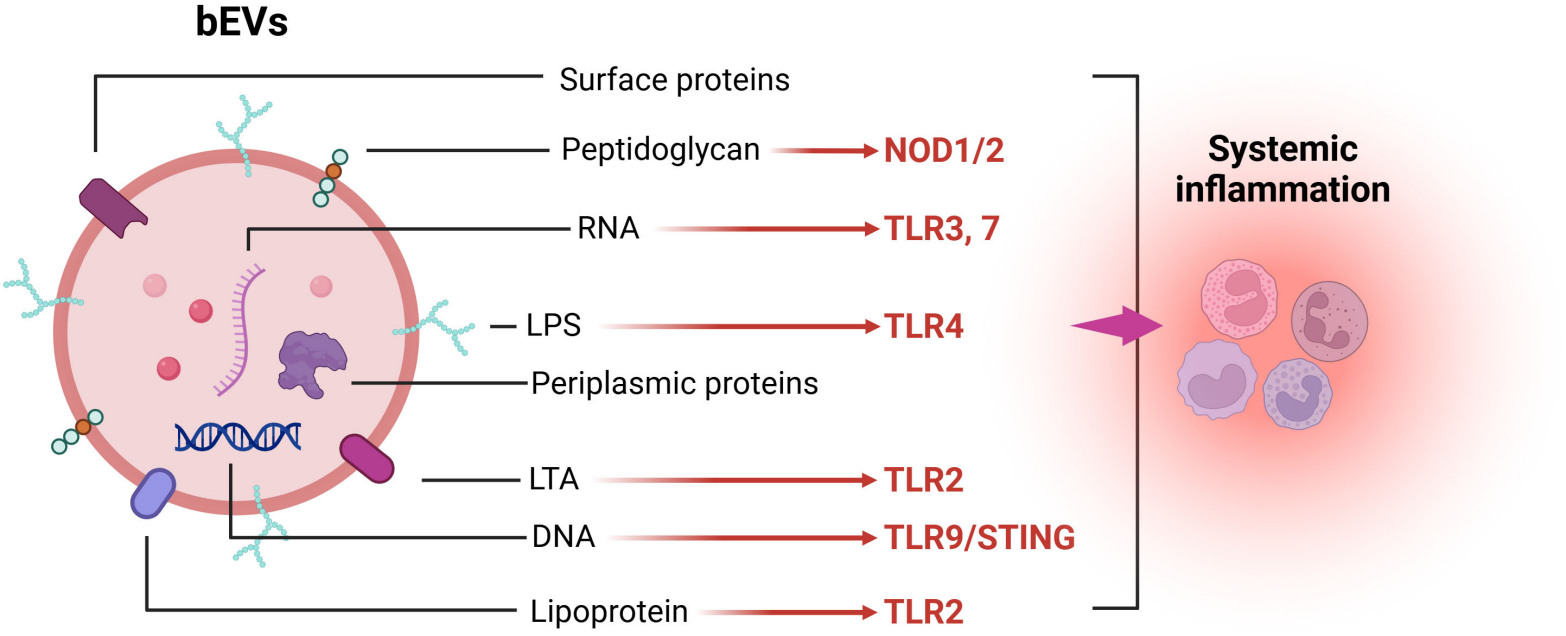
bEVs components and their pattern recognition receptors (PRRs).

Mechanistically, gram-positive and gram-negative bacteria can release bEVs carrying LTA/lipoproteins and LPS, which activate membrane TLR2 and TLR4, resulting in MyD88 and subsequent NF-κB activation, thereby promoting the production of various pro-inflammatory cytokines and chemokines (119, 120). Nucleic acids protected from degradation by bEVs further induce a potent innate immune response by activating endosomal TLR3, TLR7, and TLR9 signaling pathways. Additionally, the stimulator of interferon genes (STING) signaling pathway in the cytoplasm is triggered by DNA carried by bEVs, which further promotes the production of inflammatory factors (97). Once bEVs enter the cells, intracellular receptors such as NOD1 and NOD2 can enhance NF-κB and subsequent IL-6 and IL-8 expression through peptidoglycan activation (121, 122) (**Figure 1**). Moreover, bEVs within cells can expose bacterial components such as LPS using proteins like GBPs, SNX-10, and Hemolysin to inflammasomes NLRP3/NLRC4, thereby activating caspase-1 and caspase-11 to enhance the release of pro-inflammatory factors (123–126).

### 4.2 The role of bEVs in promoting adaptive immune responses

Recent studies have elucidated the multifaceted roles of bacterial extracellular vesicles (bEVs) in modulating immune responses, emphasizing their potential in both innate and adaptive immunity. For instance, bEVs can induce an innate immune response and are recognized by antigen-presenting cells, thereby initiating adaptive immune responses. It has been demonstrated that bEVs from *E. coli* strains can promote the production of plasma IgG, IgA, and IgM and induce a greater proportion of spleen Tc, NK, and NKT cells in healthy suckling rats (111). Additionally, dietary influences on gut microbiota have been shown to affect bEV release, evidenced by increased bEV production in mice fed a high-protein diet, which in turn promotes the expression of immunoglobulin A (IgA)-inducing cytokines and chemokines, thereby enhancing mucosal immunity (112). bEVs can also enter Peyer’s patches and activate immune cells through direct contact. Studies have shown that *Akkermansia muciniphila (A. muciniphila)* bEVs in the gut lumen can enter Peyer’s patches, activate dendritic cells (DCs), and promote their proliferation. Consequently, with the assistance of DCs, CD69^+^ B cells and IgA^+^ secreting plasma cells significantly increase, thereby elevating the concentration of intestinal IgA (113, 127). This process is crucial for reducing pathogenic microorganisms in the gut. The immunomodulatory effects of bEVs from intestinal *E. coli* strains also underscore their role in defining specific T-helper cell responses. For example, bEVs from the probiotic *E. coli Nissle 1917* program dendritic cells (DCs) to drive the pro-inflammatory Th1 response, which is essential for pathogen eradication (114). *P. gingivalis* and *Treponema denticola* bEVs stimulate dendritic cells (DCs) to induce TH17 and TH1 cell differentiation, respectively, a process potentially mediated by the increased secretion of IL-6 and IL-12 (115).

In the field of tumor therapy, *Escherichia coli*-derived bEVs have been shown to target and accumulate in tumor tissues, inducing the production of the anti-tumor cytokine CXCL10 and interferon-gamma (IFN-γ) and exerting their anti-tumor effects in an IFN-γ-dependent manner. Mechanistically, trypsin-treated bEVs failed to induce IFN-γ production, indicating that surface proteins are critical factors in inducing IFN-γ generation (116). Additionally, studies suggest that the expansion of Vγ9Vδ2 T cells induced by bEVs could represent a significant mechanism driving their anti-tumor effects (128). These findings collectively underscore the critical role of bEVs in shaping immune responses, offering promising avenues for therapeutic interventions. The pro-inflammatory effects of bEVs on the adaptive immune response are summarized in **Table 2**.

## 5 The pathogenic role of bEVs in RA

Multi-omics analyses of fecal, serum, and synovial fluid samples from RA patients have demonstrated that the gut microbiota can participate in the onset and progression of RA through invasion and metabolite secretion. Under normal circumstances, pathogenic microbes are primarily restricted by the mucosal barrier and rarely translocate into distant organs intercellularly; their primary mode of influence is through the secretion of bEVs, proteins, and metabolites. As critical players in the cross-domain interactions between the host and microbes within pathogenic environments, there is increasing evidence that bEVs participate in the host’s immune regulation by carrying molecules such as peptidoglycans, lipids, proteins, and nucleic acids (129). The presence of bEVs identified in various human biofluids and tissues suggests that these vesicles may contribute to the onset of tissue inflammation under pathological conditions (113, 130, 131).

In patients with RA, studies have identified a significant enrichment of *F. nucleatum* in fecal samples, with the levels being significantly positively correlated with RA disease activity. The bEVs secreted by *F. nucleatum* can promote the release of pro-inflammatory cytokines interleukin-8 (IL-8) and tumor necrosis factor (TNF) in colonic epithelial cells through a TLR4-dependent mechanism (132). Additionally, these bEVs can induce epithelial cell death through the FADD-RIPK1-caspase-3 signaling pathway, contributing to the development of intestinal inflammation (133). Furthermore, *F. nucleatum* bEVs can drive macrophage polarization toward the pro-inflammatory M1 phenotype (134). Interestingly, studies have demonstrated that bEVs secreted by certain gut bacteria can reach synovial tissues, confirming the possible existence of the “gut-joint axis”. Notably, bEVs derived from can migrate to the joints and trigger local inflammatory responses through the virulence factor FadA they carry. Mechanistically, these bEVs can activate the Rab5a-YB-1 signaling axis via FadA, thereby promoting the generation of synovial macrophages and the production of IL-6 and TNF-α (23, 135). These findings suggest that bacterial extracellular vesicles (bEVs) from the gut may cross the mucosal barrier and influence arthritis inflammation through the “gut-joint axis”.

As an oral pathogen closely related to the onset and development of RA, DNA components of *P. gingivalis* were identified in the synovial fluid of RA patients. However, the presence of *P. gingivalis* bacteria has not been observed (53). Interestingly, it has been confirmed that bEVs secreted by *P. gingivalis* can destroy the intestinal barrier (136). These bEVs, which transport bacterial DNA and associated effector molecules, may induce systemic inflammation and facilitate the leakage of intestinal substances into the bloodstream. This mechanism contributes to the pathogenesis of diverse systemic disorders associated with barrier dysfunction (137, 138). It was demonstrated that the ratio of cells to outer membrane vesicles (bEVs) is approximately 1:2,000. Their increased stability is attributed to their resistance to proteases and nucleases. In contrast to the original *P. gingivalis* source, bEVs demonstrate an enhanced capability to infiltrate deep tissues and elicit an inflammatory response within the host organism (139). Fleetwood and colleagues discovered that *P. gingivalis* bEVs can penetrate gingival tissue, resulting in tissue damage and inflammation. Stimulation of macrophages by bEVs resulted in the production of a significant amount of inflammatory factors, such as TNFα, IL-12p70, IL-6, IFNβ, and nitric oxide, compared to cells infected with *P. gingivalis* (140).

It was demonstrated that *P. gingivalis*’s unique PPAD can citrullinate human proteins, and contributing to the loss of tolerance to citrullinated proteins in RA (57, 141). PPAD not only disrupts amino acid balance but also compromises the body’s immune system, leading to an increased production of citrullinated antibodies against self-antigens (142). Interestingly, studies have indicated that PPAD is primarily released extracellularly through EVs (143). Moreover, PPAD in *P. gingivalis* bEVs with A-LPS modification can be protected from proteolytic degradation, which is closely associated with the citrullination of proteins (144). Notably, 78 citrullinated proteins have been identified in bEVs derived from the *P. gingivalis* W83 strain, suggesting a significant association between bEVs and RA (145). Additionally, *P. gingivalis* bEVs have been demonstrated to stimulate IL-6 and IL-8 secretion in epithelial cells via MAPK and STING pathways (97). *P. gingivalis* bEVs can also activate neutrophils, inducing degranulation without causing their death, thereby promoting inflammation (146).

**Figure 2:**
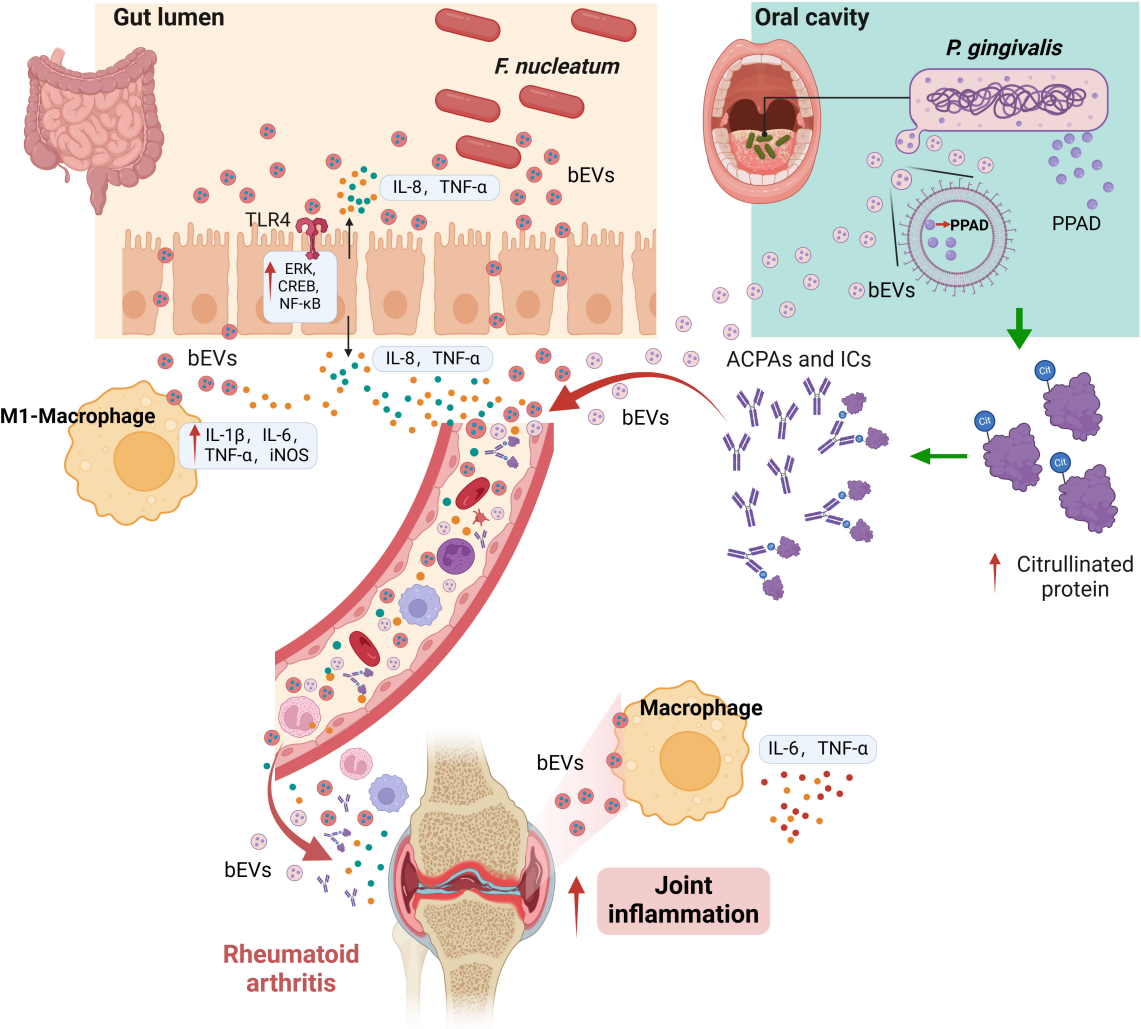
Proposed pathogenic roles of bEVs in RA. bEVs from *F. nucleatum* in the intestinal tract may enhance pro-inflammatory cytokine release and macrophage polarization, potentially influencing joint inflammation via the gut-joint axis. *P. gingivalis* bEVs, carrying PPAD, are hypothesized to penetrate mucosal barriers, thereby promoting systemic inflammation and autoimmunity by altering immune responses and protein citrullination. These mechanisms are based on emerging evidence and require further experimental validation.

Collectively, these findings suggest that bacterial extracellular vesicles (bEVs) from bacteria such as *F. nucleatum* and *P. gingivalis* exert a significant pro-inflammatory effect, potentially serving as crucial pathogenic factors in the occurrence and progression of RA (**Figure 2**).

## 6 Current challenges and future perspectives

The role of bacterial factors in inflammatory diseases is being increasingly emphasized. Changes in the abundance of bacteria such as *Prevotella copri* in the gut, as well as infections of pathogenic bacteria like *P. gingivalis* and *Aggregatibacter actinomycetemcomitans* in the oral cavity, are considered high-risk and causative factors in the onset of RA. Furthermore, recent studies have also highlighted the crucial role of the gut microbiota in modulating immune responses and contributing to the pathogenesis of other autoimmune diseases (ADs). The gut bacterium *Ruminococcus gnavus*, enriched in SLE, has been correlated with disease activity and lupus nephritis through its ability to induce the production of anti-double-stranded DNA antibodies. Additionally, specific gut commensals, such as segmented filamentous bacteria (SFB) and members of the *Erysipelotrichaceae* family, promote autoimmune inflammation by driving the polarization of T helper 17 (TH17) cells via mechanisms involving serum amyloid A (SAA) and interleukin-23 (IL-23) (147). Mechanistically, bacteria that promote the onset and progression of autoimmune diseases can induce inflammation by altering host metabolism, employing molecular mimicry, and disrupting intestinal permeability. These findings underscore the concept that the gut microbiota not only regulates local intestinal immunity but also exerts significant systemic effects by enabling gut-primed immune cells to migrate to extra-intestinal tissues, thereby exacerbating disease activity.

Emerging evidence suggests a potential role of bacterial migration from the oral cavity to the gut, forming an “oral-gut axis” which could play a synergistic role in the pathogenesis of autoimmune diseases (148). For example, oral pathogens such as *P. gingivalis* can alter the gut microbiome, leading to elevated serum endotoxin levels, increased inflammatory markers, and impaired gut barrier function, ultimately exacerbating arthritis in collagen-induced arthritis (CIA) mice. This interaction underscores the importance of studying the oral-gut axis to elucidate the intricate mechanisms underlying the connection between microbial dysbiosis and rheumatoid arthritis (RA) (149). However, whether these bacteria can reach peripheral organs such as joints through the peripheral circulation, thereby exacerbating arthritis, remains unclear. While some studies have isolated potentially pathogenic bacteria from late-stage joint fluid in RA patients (24), other studies have confirmed the absence of intact bacterial cells in the joints. Therefore, further and more in-depth research is needed to elucidate the exact pathogenic mechanisms of microbial factors in RA.

As research into eukaryotic exosomes advances, additional studies suggest that bacterial extracellular vesicles (bEVs) play a pivotal role in microbe-host cell interactions and disease development. These bEVs have also been shown to regulate both innate and adaptive immune responses, influencing immune reactions associated with RA inflammation. Hence, the discovery of bEVs may offer new insights into how pathogenic microorganisms contribute to and exacerbate inflammatory diseases such as RA. Nevertheless, because the gut and oral cavities harbor a vast array of bacterial species, bEVs derived from diverse microbial communities exhibit tremendous heterogeneity in their molecular cargo and biological activities. This diversity complicates attributing explicit pathogenic or protective functions to bEVs from any single species without detailed molecular profiling. Accordingly, clarifying how bEVs populations vary across different microbial ecosystems—both in healthy individuals and RA patients—would be instrumental in discerning their distinct effects on disease progression. In addition, despite notable progress in bEVs research, the field lacks standardized guidelines akin to the “minimal information for studies of extracellular vesicles” (MISEV) used for eukaryotic vesicles. This gap leads to varied isolation methods, impacting bEVs populations and complicating data interpretation. Moreover, the biogenesis of bEVs from bacteria remains under investigation. Although evidence supports regulated bEVs formation, the processes behind bEVs production and cargo selection remain incompletely understood. Further research is imperative to deepen our understanding of bEVs roles in inter-bacterial and bacteria-host communication.

Although the direct experimental evidence linking bEVs to RA pathogenesis remains limited, the accumulating findings from related fields strongly suggest their potential significance in autoimmune diseases, including RA. These observations support the hypothesis that bEVs may play a crucial role in RA through mechanisms such as immune cell activation, cytokine production, and antigen presentation. As mentioned above, bEVs from *P. gingivalis* have been shown to carry citrullinated proteins and PPAD, which can disrupt immune tolerance and promote the production of anti-citrullinated protein antibodies (ACPAs). Similarly, bEVs from gut bacteria such as *F. nucleatum* have been implicated in gut barrier disruption and systemic inflammation, further supporting their potential role in RA pathogenesis. However, further experimental studies are urgently needed to validate these hypotheses and better understand the specific pathways through which bEVs contribute to RA development. Future research should focus on elucidating how bEVs interact with immune cells and tissues within the RA context. For instance, experimental approaches could investigate the cargo composition of RA-specific bEVs, their uptake by synovial macrophages, and their ability to modulate T-cell responses. Additionally, studies exploring the systemic dissemination of bEVs and their impact on joint inflammation could provide critical insights into the “gut-joint” and “oral-joint” axes. By addressing these gaps, future research could establish a more comprehensive understanding of bEVs as potential mediators and therapeutic targets in RA.
